# eDNA–Amyloid Synergistic Interactions in Bacterial Biofilms: A Hidden Driver of Antimicrobial Resistance

**DOI:** 10.3390/ijms262412075

**Published:** 2025-12-15

**Authors:** Weichen Gong, Xuefei Cheng, Julio Villena, Haruki Kitazawa

**Affiliations:** 1 Laboratory of Animal Food Function, Graduate School of Agricultural Science, Tohoku University, Sendai 980-8572, Japan; 2 Livestock Immunology Unit, International Education and Research Center for Food and Agricultural Immunology (CFAI), Graduate School of Agricultural Science, Tohoku University, Sendai 980-8572, Japan; 3 Department of Microbiology and Immunology, Nihon University School of Dentistry at Matsudo, Chiba 271-8587, Japan; 4 Laboratory of Respiratory Immunology (LaRI), Division of Animal Immunology and Omics, International Education and Research Center for Food and Agricultural Immunology (CFAI), Graduate School of Agricultural Science, Tohoku University, Sendai 980-8572, Japan

**Keywords:** biofilms, extracellular DNA, amyloid proteins, antimicrobial resistance

## Abstract

Bacterial biofilms are critical contributors to chronic infections and antimicrobial resistance. Among the diverse extracellular matrix components, extracellular DNA (eDNA) and amyloid proteins have recently emerged as pivotal structural and functional molecules. Both individually contribute to biofilm stability and antibiotic tolerance, yet their cooperative roles remain underappreciated. This review aims to summarize current knowledge on the origins and functions of eDNA and amyloid proteins in biofilms, to highlight their molecular interactions, and to discuss how their synergistic effects promote biofilm-mediated resistance to antimicrobial agents. A comprehensive literature search was conducted using PubMed, Scopus, and Web of Science databases up to September 2025. Keywords included “biofilm”, “extracellular DNA”, “amyloid proteins”, “matrix”, and “antimicrobial resistance”. Relevant original research and review articles were systematically screened and critically analyzed to integrate emerging evidence on eDNA–amyloid interactions in bacterial biofilms. Current studies demonstrate that eDNA originates primarily from autolysis, active secretion, and host-derived DNA, while amyloid proteins are produced by multiple bacterial species, including *Escherichia coli* (curli), *Pseudomonas aeruginosa* (Fap), *Bacillus subtilis* (TasA), and *Staphylococcus aureus* (phenol-soluble modulins). Both molecules independently strengthen biofilm integrity and provide protective functions against antimicrobial agents. Importantly, recent evidence shows that eDNA can act as a nucleation template for amyloid fibrillation, while amyloid fibers stabilize and protect eDNA from degradation, creating a dense extracellular network. This synergistic eDNA–amyloid assembly enhances biofilm robustness, impedes antibiotic penetration, sequesters antimicrobial peptides, protects persister cells, and facilitates horizontal gene transfer of resistance determinants. The interplay between eDNA and amyloid proteins represents a central but underexplored mechanism driving biofilm-mediated antimicrobial resistance. Understanding this cooperative network not only deepens our mechanistic insights into bacterial pathogenesis but also highlights novel therapeutic targets. Strategies that disrupt eDNA–amyloid interactions may offer promising avenues for combating persistent biofilm-associated infections.

## 1. Introduction

Bacterial biofilms are highly organized multicellular communities embedded in a self-produced extracellular matrix, which provides a protective niche for microbial survival under hostile environments. Biofilms represent a formidable clinical challenge due to their intrinsic tolerance to antibiotics and resistance to host immune clearance. These microbial communities are embedded in a self-produced extracellular matrix composed of polysaccharides, proteins, lipids, and nucleic acids [1]. Among these components, extracellular DNA (eDNA) has emerged as a central player in biofilm development and maintenance [2]. Over the past decade, research has revealed that eDNA is not a passive byproduct of cell lysis but a functional molecule influencing microbial communication, stress adaptation, and antimicrobial resistance (AMR) [3]. On the other hand, amyloid proteins are another key matrix component and are widely conserved among bacteria. Bacterial amyloids have been increasingly implicated in antimicrobial resistance [4]. By forming dense fibrous networks, amyloids reduce the penetration of antibiotics, bind and neutralize cationic antimicrobial peptides (AMPs), and create protective microenvironments that favor the survival of persister cells [5,6,7]. Moreover, amyloid fibers can modulate host–pathogen interactions by masking bacterial surfaces from immune recognition and resisting proteolytic degradation, further contributing to persistence during infections [5].

Importantly, eDNA can serve as a nucleation template that accelerates amyloid fibrillation, while amyloid fibers in turn stabilize eDNA by shielding it from nuclease-mediated degradation [8]. These studies indicate that eDNA and amyloid proteins interact synergistically. eDNA can promote amyloid fibrillation, while amyloid fibers stabilize eDNA and protect it from enzymatic degradation. Together, they form a dense extracellular network that reinforces biofilm structure, impedes antibiotic penetration, sequesters antimicrobial agents, and enhances bacterial persistence.

This review summarizes current knowledge on the origins and functions of eDNA and amyloid proteins in biofilms, highlights their molecular interactions, and discusses how their synergistic roles contribute to biofilm-mediated antimicrobial resistance. Finally, we explore potential strategies to target eDNA–amyloid networks as novel therapeutic approaches against persistent bacterial infections.

## 2. eDNA in Biofilms

**Figure 1:**
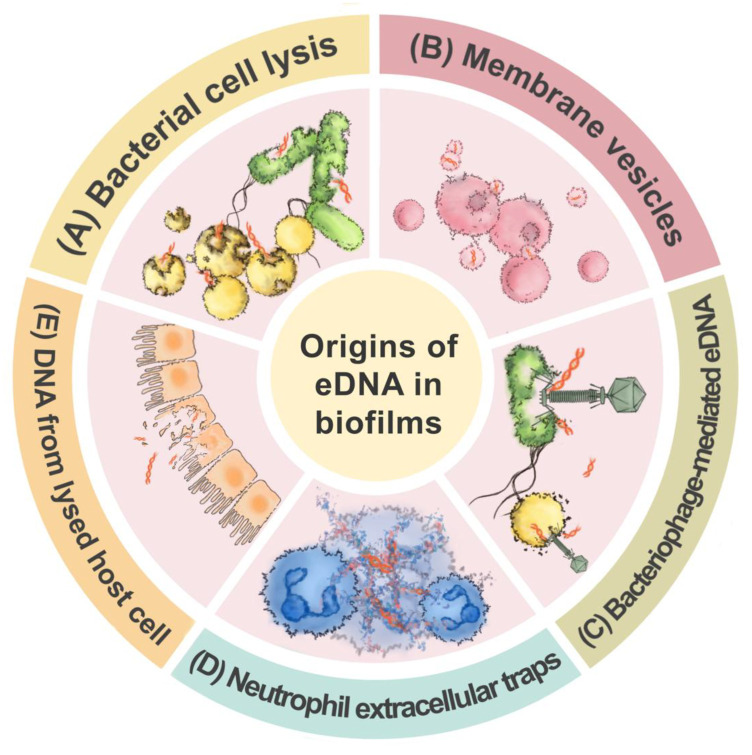
Origins of eDNA in biofilms. eDNA in biofilms mainly originates from five sources: bacterial cell lysis, secretion via membrane vesicles, bacteriophage-mediated DNA release, neutrophil extracellular traps (NETs), and DNA from lysed host cells.

The origin of eDNA in biofilms is multifactorial and depends on both intrinsic microbial processes and extrinsic environmental or host-derived factors [9]. One of the major sources of eDNA is bacterial cell lysis, which may occur via programmed autolysis or fratricidal mechanisms [10]. Collectively, eDNA within biofilms is derived from a complex interplay of bacterial autolysis, active secretion, phage-mediated lysis, and host cell disruption [3]. These diverse and overlapping sources contribute to the structural integrity, adaptive capacity, and antimicrobial tolerance of biofilms in both environmental and clinical settings (Figure 1).

### 2.1. Origin of eDNA in Biofilm

Given that eDNA release in biofilms depends more on the physiological state and environmental conditions of bacterial communities than on their taxonomic classification, a genus-based summary would not accurately capture this complexity. Therefore, we next describe the principal mechanistic routes through which eDNA is generated in biofilms, including bacterial autolysis, bacterial secretion, phage-mediated lysis, and host cell disruption.

#### 2.1.1. Bacterial Autolysis

In *Staphylococcus aureus*, autolysis is mediated by murein hydrolases such as Atl, whose activity is tightly regulated by the holin/antiholin-like proteins CidA and LrgA. These proteins form pores in the cytoplasmic membrane, facilitating the access of autolytic enzymes to the cell wall, thereby leading to the controlled release of genomic DNA into the biofilm matrix [11]. Similarly, in *Pseudomonas aeruginosa*, subpopulations of cells undergo lysis under the control of quorum sensing, releasing eDNA in a spatially organized manner that contributes to the development of structured architectures, such as the characteristic “mushroom”-shaped biofilm structures [12,13]. These processes not only supply DNA to the extracellular matrix but also facilitate genetic exchange among cells within the biofilm.

#### 2.1.2. Bacterial Secretion

In addition to passive release through lysis, some bacteria are capable of actively secreting DNA into their surroundings. Gram-negative bacteria can utilize membrane vesicles or dedicated secretion systems, such as the type IV secretion system, to extrude DNA without compromising cell viability [14,15]. Notably, although active DNA secretion was traditionally considered a feature of Gram-negative species, recent studies have demonstrated that Gram-positive bacteria such as *Streptococcus mutans* can also release eDNA through membrane vesicles [16]. This suggests that active eDNA secretion may be a conserved and underappreciated mechanism across diverse bacterial phyla.

#### 2.1.3. Phage-Mediated Lysis

Another additional and often underappreciated mechanism of eDNA release is bacteriophage-mediated lysis. During phage infection, lytic phages produce holins and endolysins that disrupt the bacterial membrane and peptidoglycan layer, leading to cell rupture and the release of intracellular contents, including genomic DNA [17,18]. This process has been shown to significantly contribute to eDNA availability in biofilms [19]. For instance, in *P. aeruginosa*, the filamentous prophage Pf4 mediates selective lysis of subpopulations, aiding in the accumulation of eDNA and maturation of biofilm architecture [20]. Similar phage-dependent lysis mechanisms have been observed in *S. aureus*, where prophage activation promotes eDNA release and supports biofilm resilience [21,22,23,24].

#### 2.1.4. Host Cell Disruption

In in vivo environments, the eDNA found within biofilms is not exclusively of bacterial origin. Host-derived DNA, particularly from neutrophil extracellular traps (NETs), contributes substantially to the DNA pool within biofilms formed during infection. Neutrophils, upon activation by microbial products, release chromatin in the form of NETs, which entangle bacteria and contribute to the biofilm scaffold [25,26,27]. Additionally, bacterial cytolytic toxins, such as pneumolysin in *Streptococcus pneumoniae* or α-toxin in *S. aureus*, can lyse host cells, releasing nuclear and mitochondrial DNA into the extracellular space [28,29]. This host-derived DNA is then incorporated into the biofilm matrix, enhancing its structural integrity and resistance to immune-mediated clearance [30].

### 2.2. Function of eDNA in Antimicrobial Resistance

One of the principal mechanisms by which eDNA contributes to resistance is through its ability to function as a physical barrier [30]. Due to its polyanionic nature, eDNA effectively binds and sequesters positively charged antibiotics, such as aminoglycosides and glycopeptides [2,31]. In *P. aeruginosa* biofilms, the incorporation of exogenous DNA significantly increases resistance to aminoglycosides, whereas mutants lacking eDNA display heightened susceptibility unless exogenous DNA is reintroduced [31]. Similarly, in *S. aureus* biofilms, eDNA serves to immobilize antibiotics, thereby limiting their diffusion and reducing their bactericidal effectiveness [32].

In addition to serving as a passive barrier, eDNA plays an active role in modulating bacterial physiology to promote resistance. By chelating divalent cations such as magnesium and calcium, eDNA mimics conditions of cation limitation and activates two-component regulatory systems such as PhoPQ and PmrAB in *P. aeruginosa* [33,34]. Activation of these pathways results in lipid A modifications that reduce outer membrane permeability and confer enhanced resistance to polymyxins and aminoglycosides, with resistance levels increasing up to eightfold [35]. Moreover, eDNA-induced magnesium depletion can trigger the Type VI Secretion System, enhancing bacterial competitiveness in polymicrobial environments and further promoting persistence during infection [36].

Structurally, eDNA contributes to the mechanical stability of the biofilm matrix by interacting with extracellular polysaccharides such as Pel and Psl. This cross-linking activity is essential for maintaining biofilm integrity, especially during early development [37,38]. Enzymatic degradation of eDNA using DNase I disrupts biofilm formation and significantly reduces biofilm biomass in both early and mature biofilms of various species, including *P. aeruginosa* and *S. aureus* [39]. The combined use of DNase I with reducing agents such as glutathione further potentiates antibiotic efficacy against clinical *P. aeruginosa* isolates, underscoring the protective role of eDNA in vivo [40].

**Figure 2:**
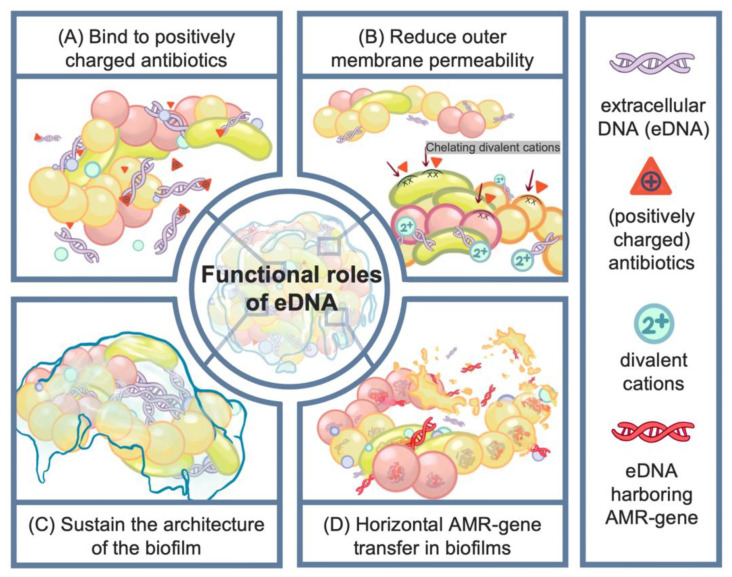
**Functional roles of eDNA in antimicrobial resistance.** eDNA can bind to positively charged antibiotics, preventing them from exerting their antimicrobial effects. In addition, eDNA decreases outer membrane permeability, further reducing antibiotic penetration. It also serves as a structural scaffold that stabilizes the biofilm matrix. Moreover, bacterial eDNA may carry antimicrobial resistance (AMR) genes, promoting horizontal gene transfer within the biofilm.

Furthermore, eDNA acts as a genetic reservoir within the biofilm, facilitating horizontal gene transfer of antibiotic resistance genes [41]. Biofilm-associated environments consistently demonstrate higher frequencies of gene exchange compared to planktonic cultures [42]. The stabilization of extracellular plasmids within the eDNA-rich matrix increases the availability of genetic material for uptake by competent cells, thereby accelerating the dissemination of resistance traits within microbial communities [43,44] (Figure 2).

## 3. Amyloid Protein in Biofilms

### 3.1. Characteristics of Bacterial Amyloid Proteins

Among the diverse extracellular components of bacterial biofilms, amyloid proteins have emerged as particularly well-studied structural and functional elements. Representative examples include curli fibers in *E. coli*, Fap fibrils in *P. aeruginosa*, TasA in *B. subtilis*, and phenol-soluble modulins (PSMs) in *S. aureus* [45,46,47,48]. These proteins have become “model systems” for studying bacterial amyloids, much as model organisms are used to investigate fundamental biological processes. Insights gained from these systems provide a framework to understand the common properties and functions of amyloid proteins across bacterial species.

A defining feature of bacterial amyloids is their ability to self-assemble into β-sheet-rich fibrils that are highly stable and resistant to proteolysis, heat, and chemical disruption [23,49]. This stability ensures their persistence in the extracellular matrix and allows them to act as long-lived scaffolds that reinforce biofilm architecture [23]. Regardless of their species of origin, amyloid fibrils share this fundamental characteristic, making them ideal structural components in the harsh and fluctuating environments where biofilms develop.

### 3.2. Function of Amyloid Protein in Antimicrobial Resistance

Several studies have demonstrated that the dense amyloid meshwork reduces the penetration of antibiotics into the biofilm interior [50]. For example, in *E. coli*, curli amyloids are associated with reduced susceptibility to β-lactams and aminoglycosides, largely due to impaired antibiotic diffusion through the fibrillar matrix [45,51]. Similarly, *B. subtilis* TasA amyloids have been shown to strengthen the extracellular scaffold, creating diffusion-limiting barriers that protect embedded cells from exposure to antimicrobial agents [52,53].

Amyloid proteins can also directly interact with AMPs. Their repetitive β-sheet surfaces and exposed charged residues enable binding of cationic AMPs [54,55]. In *S. aureus*, amyloid-like PSMs assemble into fibrils that not only stabilize the biofilm but also sequester host-derived AMPs, thereby neutralizing their bactericidal effects [56]. *P. aeruginosa* Fap fibrils have likewise been reported to bind small-molecule AMPs, decreasing their availability and activity [57].

In addition, amyloid-mediated biofilm reinforcement promotes the formation of microenvironments that favor the persistence of antibiotic-tolerant subpopulations. The compact and oxygen-limited zones within amyloid-rich regions reduce bacterial metabolic activity, a condition known to facilitate persister survival under antibiotic stress [58]. This has been observed in *E. coli* and *P. aeruginosa* biofilms, where amyloid-enriched matrices correlate with higher proportions of persister cells following antibiotic treatment [51,59].

These examples highlight that bacterial amyloids are not merely passive scaffolds but multifunctional components that actively enhance antimicrobial resistance by forming resilient fibrillar networks, binding and neutralizing antimicrobial agents, and fostering protective niches for persister cells.

## 4. eDNA–Amyloid Interactions and Their Synergistic Role in Antimicrobial Resistance

**Figure 3:**
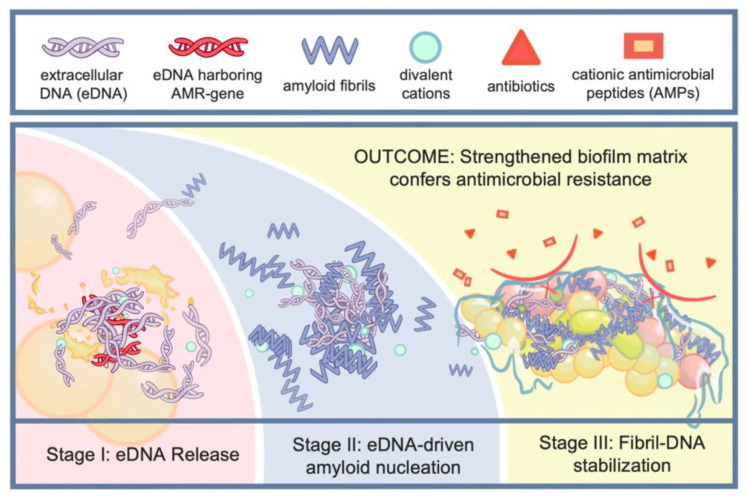
**Synergistic function of eDNA–amyloid in antimicrobial resistance.** Once released from bacteria, eDNA acts as a nucleation factor that promotes amyloid protein aggregation. This interaction strengthens the biofilm matrix, ultimately enhancing its resistance to antimicrobial agents.

eDNA and amyloid proteins act synergistically to enhance biofilm stability and antimicrobial resistance [60,61,62]. eDNA can serve as a nucleation template that accelerates amyloid fibril formation, while amyloid fibers stabilize eDNA and protect it from nuclease degradation, forming a dense and resilient extracellular network [60]. This cooperative assembly is more effective at conferring antimicrobial tolerance than either component alone. Mechanistically, polyanionic eDNA can act as a nucleation scaffold: electrostatic and hydrogen-bonding interactions between the phosphate backbone and amyloidogenic peptides orient monomers/oligomers and lower the energy barrier for β-sheet stacking, thereby accelerating nucleation and localized fiber growth [31]. Once fibrils are formed, the extended β-sheet surfaces and associated hydrophobic patches of amyloid fibers bind and sterically shield eDNA from nuclease attack, effectively prolonging the lifetime of extracellular genetic material in the matrix [33,63] (Figure 3).

Once incorporated into the matrix, DNA–amyloid complexes reinforce the biofilm in several interrelated ways. First, the intertwined fibrillar network forms a densely cross-linked architecture that slows the inward movement of many antibiotics, effectively reducing the concentration of active drug that reaches deeper bacterial subpopulations [64,65]. In addition, the strongly charged surface of DNA–amyloid composites provides extensive binding interfaces capable of capturing and immobilizing cationic antimicrobial peptides as well as certain positively charged or amphipathic antibiotics, thereby reducing their bioavailability [65]. Finally, by shaping the mechanical and spatial organization of the matrix, these composites contribute to the development of stratified microniches with altered metabolic activity. Bacteria residing in these regions often adopt slow-growing or dormant states associated with persister formation, further diminishing susceptibility to conventional antimicrobials [63].

These mechanistic principles are supported by multiple experimental systems. In *P. aeruginosa*, the interaction between eDNA and Fap amyloid fibers creates a tightly packed matrix that impedes the diffusion of antibiotics such as aminoglycosides and β-lactams [54]. The negatively charged eDNA attracts cationic AMPs, while the amyloid scaffold reinforces the matrix, collectively reducing antimicrobial access to embedded cells [66,67]. A similar synergy has been observed in *S. aureus*, where PSMs assemble into amyloid fibrils in the presence of eDNA [66]. The resulting fibril–DNA network not only strengthens the biofilm mechanically but also sequesters host-derived AMPs and protects eDNA, promoting horizontal transfer of resistance genes within the biofilm. Analogous interactions occur in *E. coli* biofilms, where curli fibers associate with eDNA to form extracellular scaffolds that resist enzymatic degradation and limit antibiotic penetration [68,69]. These dense fibril–DNA networks generate localized microenvironments with reduced oxygen and nutrient availability, favoring the survival of metabolically inactive persister cells, which are inherently tolerant to multiple classes of antibiotics [68,70]. More broadly, bacterial-derived DNA has been shown to accelerate amyloid aggregation in vitro, demonstrating that DNA from different bacterial sources can modulate nucleation and elongation kinetics of amyloidogenic proteins [71]. Additionally, amyloid–DNA composites can act as persistent reservoirs of extracellular nucleic acids that are competent for horizontal gene transfer and that stimulate host immune responses, further linking the composite material to both dissemination of resistance determinants and chronic inflammation [64].

Across these examples, a common theme emerges: eDNA–amyloid networks confer mechanical protection, chemical sequestration of antimicrobials, and physiological modulation of embedded bacteria, collectively amplifying biofilm-mediated antimicrobial resistance.

## 5. Perspectives

Understanding the synergistic interactions between eDNA and amyloid proteins in biofilms highlights new therapeutic opportunities against antimicrobial-resistant bacteria. Since the stability of the biofilm matrix depends on the mutual reinforcement between these two components, strategies that simultaneously target both may prove more effective than single-agent approaches. One promising avenue is the combination of DNases, which degrade extracellular DNA, with anti-amyloid compounds that disrupt β-sheet fibril assembly. Such a dual strategy could dismantle the composite eDNA–amyloid network, thereby restoring antibiotic penetration and increasing bacterial susceptibility. Importantly, the amyloid component—long overshadowed by nucleic acids and polysaccharides in biofilm research—represents an underexploited yet pivotal therapeutic target. Amyloid fibrils are uniquely stable, highly resistant to proteolytic degradation, and often persist even after antibiotic treatment. By focusing on amyloid disruption alongside eDNA degradation, the overall integrity of the matrix can be weakened in a sustained manner, rather than temporarily disrupted.

Beyond enzymatic and anti-amyloid interventions, additional strategies could be envisioned. For example, molecular tweezers have been shown to inhibit the formation of *S. aureus* biofilm-associated functional amyloids by selectively disrupting the electrostatic and hydrophobic interactions essential for fibril assembly, providing a promising strategy to target amyloid-based biofilm matrices [65]. Cationic peptide mimetics that outcompete eDNA for binding sites on amyloid fibrils may destabilize the network, while nanoparticle-based delivery systems can concentrate DNase or amyloid inhibitors specifically at infection sites [72,73]. Moreover, considering that eDNA–amyloid interactions promote horizontal gene transfer and persister formation, therapies that disrupt these interactions may not only sensitize biofilms to antibiotics but also reduce the long-term evolution of resistance.

Another promising research direction involves repurposing anti-amyloid agents developed for neurodegenerative diseases, such as epigallocatechin gallate (EGCG), doxycycline, and curcumin derivatives, which have demonstrated activity against bacterial amyloids in vitro [73]. For instance, EGCG can remodel preformed amyloid fibers into non-toxic, amorphous aggregates while also interfering with eDNA binding [74]. Such dual activity—modulating both amyloid and DNA—suggests that compounds originally designed for human amyloidoses might be re-engineered as anti-biofilm agents. In parallel, structure-based drug design and cryo-EM studies of bacterial amyloid proteins (e.g., CsgA, FapC, TasA, PSMs) can provide molecular blueprints for developing next-generation inhibitors that selectively target bacterial amyloid interfaces without affecting host proteins.

Looking forward, greater scientific attention must be directed toward the biology of bacterial amyloid proteins themselves—their biosynthetic pathways, secretion mechanisms, post-translational regulation, and interspecies diversity. Key questions include how amyloid expression is coordinated with eDNA release, how host factors influence amyloid assembly, and how these fibrils interact with ions, lipids, and proteins in the biofilm milieu. Addressing these questions is essential for identifying vulnerabilities that can be pharmacologically exploited.

Furthermore, omics-based and structural approaches can greatly accelerate this research. Transcriptomic and proteomic profiling during different biofilm stages can reveal co-regulation patterns between amyloid genes and nucleases, while cryo-electron microscopy and solid-state NMR can elucidate the three-dimensional structures of amyloid fibrils bound to eDNA. Such insights would clarify the physicochemical basis of eDNA–amyloid synergy and guide rational drug design. Computational modeling could also predict how disrupting this interaction alters diffusion, mechanical properties, and antibiotic gradients within biofilms, providing a quantitative framework for therapeutic optimization.

From a broader perspective, targeting the eDNA–amyloid axis represents a conceptual shift in how we approach bacterial resistance. Traditional antimicrobial therapies aim to kill planktonic cells or inhibit essential metabolic pathways, but biofilm infections persist because resistance arises from matrix-mediated protection rather than intrinsic genetic resistance. Dismantling the biofilm’s physical scaffold—by enzymatically degrading eDNA and destabilizing amyloid fibrils—attacks the structural basis of resilience itself. This strategy reframes antimicrobial resistance not merely as a cellular phenomenon but as an emergent property of microbial community organization.

From a therapeutic perspective, targeting eDNA–amyloid synergy alone may be insufficient if upstream regulatory mechanisms remain intact. Quorum sensing (QS) represents a promising complementary target, as QS inhibition can suppress both eDNA release and amyloid protein production at the population level. Biofilm development is tightly regulated by QS, a population density-dependent signaling system that coordinates collective bacterial behaviors. QS has been shown to control multiple processes essential for biofilm maturation, including extracellular polymeric substance production, cell lysis–associated eDNA release, and the expression of functional amyloid proteins. Increasing evidence suggests that QS signaling indirectly shapes the formation and stabilization of eDNA–amyloid complexes by synchronizing the availability of both nucleic acid scaffolds and amyloidogenic components within the biofilm matrix. Therefore, understanding eDNA–amyloid synergy in biofilms requires consideration of QS as an upstream regulatory layer that modulates matrix assembly and antimicrobial tolerance. We propose that a combinatorial antibiofilm strategy integrating quorum-sensing inhibitors, extracellular nucleases, and anti-amyloid agents may yield synergistic benefits. QS inhibitors can reduce matrix component production at the transcriptional level, DNases can dismantle existing eDNA scaffolds, and anti-amyloid compounds can destabilize amyloid fibrils and prevent matrix reassembly. Such a multi-pronged approach may effectively disrupt biofilm integrity, restore antimicrobial penetration, and reduce the emergence of persister cells, thereby enhancing treatment outcomes against chronic biofilm-associated infections [75].

Finally, future work should focus on synergistic treatment regimens that integrate enzymatic, chemical, and conventional antibiotic therapies. Targeting the eDNA–amyloid represents a paradigm shift from eradicating planktonic cells to dismantling the protective architecture of biofilms. Such approaches may provide a rational path to overcoming the formidable challenge of antimicrobial resistance in biofilm-associated infections.

## 6. Conclusions

Biofilm-associated antimicrobial resistance remains one of the most pressing challenges in infectious disease. Within this context, eDNA and amyloid proteins stand out as two fundamental structural components of the biofilm matrix. While each contributes independently to biofilm stability and protection, their synergistic interaction produces a composite network that is exceptionally resilient against antibiotics and host defenses. By facilitating matrix cohesion, sequestering antimicrobial agents, and supporting persister cell formation, the eDNA–amyloid plays a central role in biofilm-associated tolerance.

Recognizing the importance of this synergistic interaction offers new opportunities for intervention. Targeting both eDNA and amyloid simultaneously—through the combined use of nucleases, anti-amyloid agents, and conventional antibiotics—may represent a promising strategy to overcome the formidable defenses of biofilm-forming pathogens. As research continues to elucidate the mechanisms of eDNA–amyloid interactions, this knowledge will guide the development of innovative therapeutics aimed not merely at killing bacteria, but at dismantling the protective biofilm matrix itself.

Despite growing awareness, bacterial amyloid research remains disproportionately underdeveloped compared with other biofilm components such as polysaccharides or nucleic acids. The majority of studies have centered on *E. coli* curli, while amyloid systems in other pathogens—particularly those with major clinical relevance like *P. aeruginosa*, *S. aureus*, and *Enterococcus faecalis*—are poorly characterized. This imbalance limits our ability to generalize mechanistic insights and design broad-spectrum interventions. Future research must therefore prioritize systematic investigation of bacterial amyloid diversity, including their structural polymorphism, post-assembly stability, and interactions with extracellular nucleic acids.

In this context, targeting both eDNA and amyloids simultaneously offers a powerful and rational therapeutic framework. The combined use of nucleases, anti-amyloid agents, and optimized antibiotics could dismantle the biofilm matrix, enhance drug penetration, and restore bacterial sensitivity. Moreover, since amyloid structures are often more stable than other matrix components, their targeted disruption may yield longer-lasting therapeutic benefits and reduce biofilm recurrence. Conceptually, this approach reflects a broader scientific shift—from viewing resistance as a purely genetic trait to understanding it as a collective and structural property of microbial communities.

In summary, future biofilm and AMR research must elevate bacterial amyloid proteins from secondary curiosities to central subjects of investigation. Their unique biochemical stability, self-assembly dynamics, and cooperative interaction with eDNA make them indispensable to biofilm resilience. Understanding and targeting these proteins will not only deepen our mechanistic insight into microbial survival strategies but also provide a conceptual foundation for next-generation anti-biofilm therapeutics. The long-term solution to biofilm-mediated antimicrobial resistance lies not in simply designing stronger antibiotics, but in unraveling and disrupting the molecular architecture that allows bacteria to endure in the first place.

## Data Availability

Not applicable.
